# Aqua­[4′-(4-chloro­phen­yl)-2,2′:6′,2′′-terpyridine]­nitratocopper(II) nitrate [4′-(4-chloro­phen­yl)-2,2′:6′,2′′-terpyridine]­dinitratocopper(II) monohydrate

**DOI:** 10.1107/S1600536811005290

**Published:** 2011-02-19

**Authors:** Juan Zhao, Shuo Shi, Tianming Yao, Zhongfei Tan

**Affiliations:** a Department of Chemistry, Tongji University, Shanghai, People’s Republic of China

## Abstract

The crystal structure of the title compound, [Cu(NO_3_)(C_21_H_14_ClN_3_)(H_2_O)]NO_3_·[Cu(NO_3_)_2_(C_21_H_14_ClN_3_)]·H_2_O, con­sists of two crystallographically independent Cu^II^ complexes, in which each copper cation is penta­coordinated by three N atoms of the chelating ligand and two O atoms of nitrate anions or water mol­ecules. One of the coordinated nitrate anions is disordered over two set of sites in a 0.85:0.15 ratio.

## Related literature

For the use of substituted terpyridine ligands in coordination chemistry due to their ability to form complexes with transition metals, see: Chen *et al.* (2010 ▶); Feng *et al.* (2006 ▶); Hou *et al.* (2005 ▶); Mutai *et al.* (2001 ▶). For the synthesis of the title compound, see: Mutai *et al.* (2001 ▶). For related structures, see: Granifo *et al.* (2004 ▶); Chen *et al.* (2010 ▶). For the biochemcial importance of terpyridine ligands, see: Bertrand *et al.* (2007 ▶); Maity *et al.* (2010 ▶). 
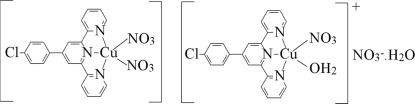


## Experimental

### 

#### Crystal data

[Cu(NO_3_)_2_(C_21_H_14_ClN_3_)][Cu(NO_3_)(C_21_H_14_ClN_3_)(H_2_O)]NO_3_·H_2_O
*M* *_r_* = 1098.76
Monoclinic, 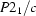

*a* = 14.7172 (2) Å
*b* = 15.0680 (2) Å
*c* = 20.4214 (3) Å
β = 105.377 (1)°
*V* = 4366.51 (10) Å^3^
*Z* = 4
Cu *K*α radiation
μ = 3.04 mm^−1^
*T* = 293 K
0.30 × 0.20 × 0.20 mm

#### Data collection

Bruker SMART CCD area-detector diffractometer
Absorption correction: multi-scan (*SADABS*; Bruker, 2000 ▶) *T*_min_ = 0.462, *T*_max_ = 0.581
17732 measured reflections
8572 independent reflections
7142 reflections with *I* > 2σ(*I*)
*R*_int_ = 0.027

#### Refinement

*R*[*F* ^2^ > 2σ(*F* ^2^)] = 0.044
*wR*(*F* ^2^) = 0.115
*S* = 1.00
8572 reflections
643 parameters
15 restraints
H-atom parameters constrained
Δρ_max_ = 0.56 e Å^−3^
Δρ_min_ = −0.27 e Å^−3^

### 

Data collection: *SMART* (Bruker, 2000 ▶); cell refinement: *SAINT* (Bruker, 2000 ▶); data reduction: *SAINT*; program(s) used to solve structure: *SHELXS97* (Sheldrick, 2008 ▶); program(s) used to refine structure: *SHELXL97* (Sheldrick, 2008 ▶); molecular graphics: *XP* in *SHELXTL* (Sheldrick, 2008 ▶); software used to prepare material for publication: *CIFTAB* in *SHELXL97*.

## Supplementary Material

Crystal structure: contains datablocks I, global. DOI: 10.1107/S1600536811005290/nc2216sup1.cif

Structure factors: contains datablocks I. DOI: 10.1107/S1600536811005290/nc2216Isup2.hkl

Additional supplementary materials:  crystallographic information; 3D view; checkCIF report

## Figures and Tables

**Fig. 1.**
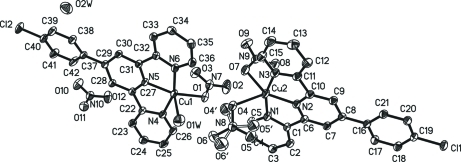
ORTEP drawing of the title compound with labelling showing displacement ellipsoids at the 30% probability level. All hydrogen atoms have been omitted for clarity.

## supplementary crystallographic information

### Comment

Substituted terpyridine ligands have attracted widespread attention in coordination chemistry due to their ability to form complexes with transition metals,  (Chen *et al.*,2010; Feng *et al.*,2006; Granifo *et al.*,2004; Hou *et al.*,2005; Mutai *et al.*,2001). Recent studies reveal that under certain conditions, copper complexes with phenanthroline and polypyridine ligands have good DNA binding activity, which attracted great interest in biochemistry (Bertrand *et al.*,2007; Maity *et al.*,2010). The crystal structure of the title compound was determined as part of an ongoing study of the properties of copper complex containing terpyridyl ligands.

The crystal structure of the title compound consists of two crystallographically independent complexes, in which the copper cations are always penta coordinated within a distorted square-pyramidal coordination (Fig.1). In one of these complexes two copper atom is coordinated by three N atoms of the tridentrate chelating ligand and two O atoms of two independent two nitrate anions.In the other complex the copper is coordinated by three N atoms of the chelating ligand, one O atom of one nitrate anion and an O atom of one water molecule. The structure contains one additional nitrate anion and one additional water molecule that are not connected to the copper cations. The central Cu–N bond lengths [Cu(1)—N(5) = 1.9287 (16) Å and Cu(2)—N(2) = 1.9256 (18)Å] are slightly shorter than those to the outer N atoms of 2.0178 (18), 2.0277 (18), 2.0096 (19) and 2.014 (2)Å. The bond angles to the N atoms of the terpyridyl unit of 80.40 (8), 80.07 (8), 80.39 (7) and 79.97 (7) ° deviate from the ideal values which is a common characteristics for terpyridyl-containing complexes (Granifo *et al.*,2004). The apical Cu–O bond lenghts (Cu(1)—O(1 W) 2.208 (2) Å and Cu(2)—O(4) 2.163 (3) Å) are longer than the other Cu–O bonds.

### Experimental

4'-(4-Chlorophenyl)-2,2':6',2''-terpyridine (Cl-ptp) has been synthesized by a procedure reported in the literature (Mutai *et al.* 2001). A solution of Cl-ptp (42.35 mg, 0.124 mmol) in dichloromethane (3 ml) was mixed with anhydrous acetonitrile (3 ml) dissolving copper nitrate (29.96 mg, 0.124 mmol),then sealed and kept it in refrigerator. Several days later, blue crystals were collected by filtration.

### Refinement

All H atom were positioned geometrically and refined as riding, with aromatic C—H =0.93 Å and with Uiso(H) = 1.2Ueq(C).The water H were located in difference map and were refined isotropic.

### Crystal data

**Table:**

[Cu(NO_3_)_2_(C_21_H_14_ClN_3_)][Cu(NO_3_)(C_21_H_14_ClN_3_)(H_2_O)]NO_3_·H_2_O	*Z* = 4
*M**_r_* = 1098.76	*F*(000) = 2232
Monoclinic, *P*2_1_/*c*	*D*_x_ = 1.671 Mg m^−^^3^
*a* = 14.7172 (2) Å	Cu *K*α radiation, λ = 1.54178 Å
*b* = 15.0680 (2) Å	µ = 3.04 mm^−^^1^
*c* = 20.4214 (3) Å	*T* = 293 K
β = 105.377 (1)°	Block, blue
*V* = 4366.51 (10) Å^3^	0.30 × 0.20 × 0.20 mm

### Data collection

**Table:**

Bruker SMART CCD area-detector diffractometer	8572 independent reflections
Radiation source: fine-focus sealed tube	7142 reflections with *I* > 2σ(*I*)
graphite	*R*_int_ = 0.027
ω scans	θ_max_ = 72.9°, θ_min_ = 3.1°
Absorption correction: multi-scan (*SADABS*; Bruker, 2000)	*h* = −18→17
*T*_min_ = 0.462, *T*_max_ = 0.581	*k* = −18→17
17732 measured reflections	*l* = −24→25

### Refinement

**Table:**

Refinement on *F*^2^	Primary atom site location: structure-invariant direct methods
Least-squares matrix: full	Secondary atom site location: difference Fourier map
*R*[*F*^2^ > 2σ(*F*^2^)] = 0.044	Hydrogen site location: inferred from neighbouring sites
*wR*(*F*^2^) = 0.115	H-atom parameters constrained
*S* = 1.00	*w* = 1/[σ^2^(*F*_o_^2^) + (0.0792*P*)^2^] where *P* = (*F*_o_^2^ + 2*F*_c_^2^)/3
8572 reflections	(Δ/σ)_max_ = 0.001
643 parameters	Δρ_max_ = 0.56 e Å^−^^3^
15 restraints	Δρ_min_ = −0.27 e Å^−^^3^

### Special details

**Table:**

Geometry. All e.s.d.'s (except the e.s.d. in the dihedral angle between two l.s. planes) are estimated using the full covariance matrix. The cell e.s.d.'s are taken into account individually in the estimation of e.s.d.'s in distances, angles and torsion angles; correlations between e.s.d.'s in cell parameters are only used when they are defined by crystal symmetry. An approximate (isotropic) treatment of cell e.s.d.'s is used for estimating e.s.d.'s involving l.s. planes.
Refinement. Refinement of *F*^2^ against ALL reflections. The weighted *R*-factor *wR* and goodness of fit *S* are based on *F*^2^, conventional *R*-factors *R* are based on *F*, with *F* set to zero for negative *F*^2^. The threshold expression of *F*^2^ > σ(*F*^2^) is used only for calculating *R*-factors(gt) *etc*. and is not relevant to the choice of reflections for refinement. *R*-factors based on *F*^2^ are statistically about twice as large as those based on *F*, and *R*- factors based on ALL data will be even larger.

### Fractional atomic coordinates and isotropic or equivalent isotropic displacement parameters (Å^2^)

**Table:**

	*x*	*y*	*z*	*U*_iso_*/*U*_eq_	Occ. (<1)
Cu2	0.37161 (3)	0.70822 (2)	0.243697 (17)	0.04814 (11)	
Cl1	−0.06351 (4)	0.37787 (4)	−0.19024 (3)	0.05263 (14)	
N1	0.38825 (15)	0.58174 (12)	0.27589 (10)	0.0443 (4)	
N2	0.30021 (13)	0.64907 (11)	0.16278 (9)	0.0395 (3)	
N3	0.32876 (16)	0.81395 (13)	0.18301 (11)	0.0499 (4)	
N8	0.57336 (15)	0.69059 (13)	0.25635 (10)	0.0481 (4)	
O4	0.5200 (2)	0.73693 (19)	0.28333 (17)	0.0547 (6)	0.85
O5	0.5407 (3)	0.6533 (3)	0.20330 (17)	0.0886 (10)	0.85
O6	0.6581 (2)	0.6877 (3)	0.28725 (18)	0.0811 (8)	0.85
O4'	0.5462 (19)	0.7293 (19)	0.2997 (11)	0.085 (9)*	0.15
O5'	0.5103 (12)	0.6818 (14)	0.2011 (8)	0.074 (5)*	0.15
O6'	0.6530 (13)	0.6649 (19)	0.2589 (15)	0.107 (8)*	0.15
N9	0.28984 (17)	0.76429 (13)	0.34439 (11)	0.0520 (5)	
O7	0.36819 (15)	0.77450 (13)	0.33053 (10)	0.0605 (5)	
O8	0.23158 (17)	0.71330 (18)	0.30946 (15)	0.0806 (7)	
O9	0.2733 (2)	0.80703 (15)	0.39136 (12)	0.0764 (6)	
C1	0.34510 (15)	0.52113 (14)	0.22914 (10)	0.0380 (4)	
C2	0.35475 (17)	0.43099 (14)	0.24211 (11)	0.0439 (4)	
H2A	0.3244	0.3898	0.2097	0.053*	
C3	0.41109 (19)	0.40344 (16)	0.30496 (12)	0.0492 (5)	
H3A	0.4191	0.3433	0.3149	0.059*	
C4	0.45462 (19)	0.46552 (18)	0.35206 (12)	0.0517 (5)	
H4A	0.4924	0.4480	0.3942	0.062*	
C5	0.44127 (19)	0.55490 (17)	0.33570 (12)	0.0516 (5)	
H5A	0.4704	0.5971	0.3676	0.062*	
C6	0.29006 (14)	0.56106 (13)	0.16427 (10)	0.0372 (4)	
C7	0.23287 (15)	0.51691 (12)	0.10898 (10)	0.0381 (4)	
H7A	0.2253	0.4557	0.1103	0.046*	
C8	0.18654 (14)	0.56533 (13)	0.05104 (10)	0.0368 (4)	
C9	0.19961 (16)	0.65762 (14)	0.05147 (11)	0.0415 (4)	
H9A	0.1702	0.6916	0.0138	0.050*	
C10	0.25694 (16)	0.69735 (13)	0.10885 (11)	0.0399 (4)	
C11	0.27548 (17)	0.79410 (13)	0.11984 (12)	0.0442 (4)	
C12	0.2395 (2)	0.85883 (15)	0.07258 (14)	0.0528 (5)	
H12A	0.2039	0.8440	0.0292	0.063*	
C13	0.2579 (2)	0.94744 (16)	0.09149 (17)	0.0619 (7)	
H13A	0.2340	0.9926	0.0607	0.074*	
C14	0.3114 (2)	0.96745 (16)	0.15558 (18)	0.0647 (7)	
H14A	0.3240	1.0262	0.1689	0.078*	
C15	0.3459 (2)	0.89932 (17)	0.19996 (16)	0.0606 (6)	
H15A	0.3825	0.9130	0.2433	0.073*	
C16	0.12483 (14)	0.51953 (12)	−0.00913 (10)	0.0357 (4)	
C17	0.11446 (17)	0.42722 (14)	−0.00964 (11)	0.0435 (4)	
H17A	0.1471	0.3945	0.0278	0.052*	
C18	0.05659 (18)	0.38366 (13)	−0.06473 (12)	0.0457 (5)	
H18A	0.0500	0.3223	−0.0642	0.055*	
C19	0.00875 (15)	0.43232 (14)	−0.12038 (10)	0.0395 (4)	
C20	0.01727 (17)	0.52367 (14)	−0.12187 (11)	0.0458 (5)	
H20A	−0.0155	0.5560	−0.1596	0.055*	
C21	0.07546 (17)	0.56623 (13)	−0.06619 (11)	0.0446 (5)	
H21A	0.0816	0.6276	−0.0670	0.053*	
Cu1	0.64469 (2)	0.755439 (18)	0.511420 (14)	0.03832 (10)	
Cl2	1.01554 (6)	1.13769 (4)	0.93957 (3)	0.0688 (2)	
O1	0.53539 (13)	0.68233 (11)	0.46355 (8)	0.0514 (4)	
O1W	0.71041 (19)	0.71742 (18)	0.43010 (11)	0.0773 (6)	
H1O1	0.6778	0.7146	0.3890	0.116*	
H2O1	0.7678	0.7228	0.4297	0.116*	
O2	0.4079 (2)	0.6278 (2)	0.48005 (15)	0.0932 (9)	
O3	0.48744 (19)	0.72562 (16)	0.54977 (12)	0.0739 (6)	
N4	0.71391 (13)	0.65875 (11)	0.57323 (9)	0.0393 (3)	
N5	0.71651 (12)	0.82709 (11)	0.58543 (8)	0.0345 (3)	
N6	0.60310 (13)	0.87827 (11)	0.47501 (9)	0.0394 (3)	
N7	0.47370 (16)	0.67908 (14)	0.49853 (11)	0.0536 (5)	
C22	0.77267 (14)	0.68840 (12)	0.63225 (10)	0.0356 (4)	
C23	0.82755 (17)	0.63125 (14)	0.67921 (11)	0.0443 (4)	
H23A	0.8669	0.6526	0.7196	0.053*	
C24	0.8226 (2)	0.54068 (15)	0.66470 (14)	0.0533 (5)	
H24A	0.8593	0.5007	0.6952	0.064*	
C25	0.7630 (2)	0.51087 (14)	0.60480 (14)	0.0547 (6)	
H25A	0.7591	0.4507	0.5943	0.066*	
C26	0.70929 (18)	0.57172 (15)	0.56072 (12)	0.0484 (5)	
H26A	0.6682	0.5513	0.5207	0.058*	
C27	0.77161 (14)	0.78655 (12)	0.63989 (10)	0.0343 (4)	
C28	0.82017 (14)	0.83482 (13)	0.69578 (10)	0.0363 (4)	
H28A	0.8594	0.8064	0.7331	0.044*	
C29	0.80945 (14)	0.92764 (12)	0.69537 (10)	0.0353 (4)	
C30	0.74968 (15)	0.96743 (12)	0.63802 (10)	0.0367 (4)	
H30A	0.7402	1.0285	0.6366	0.044*	
C31	0.70481 (14)	0.91482 (12)	0.58339 (10)	0.0347 (4)	
C32	0.64043 (14)	0.94527 (13)	0.51785 (10)	0.0351 (4)	
C33	0.62025 (15)	1.03327 (13)	0.50081 (10)	0.0384 (4)	
H33A	0.6463	1.0784	0.5310	0.046*	
C34	0.55991 (16)	1.05270 (14)	0.43731 (11)	0.0427 (4)	
H34A	0.5448	1.1112	0.4246	0.051*	
C35	0.52309 (16)	0.98441 (16)	0.39380 (11)	0.0465 (5)	
H35A	0.4834	0.9964	0.3510	0.056*	
C36	0.54549 (17)	0.89771 (15)	0.41408 (11)	0.0452 (4)	
H36A	0.5198	0.8517	0.3846	0.054*	
C37	0.86092 (15)	0.98066 (13)	0.75508 (10)	0.0376 (4)	
C38	0.87342 (17)	1.07184 (14)	0.75065 (11)	0.0447 (5)	
H38A	0.8495	1.1006	0.7094	0.054*	
C39	0.9212 (2)	1.12044 (14)	0.80709 (13)	0.0516 (5)	
H39A	0.9288	1.1814	0.8037	0.062*	
C40	0.95691 (18)	1.07797 (15)	0.86793 (11)	0.0473 (5)	
C41	0.9460 (2)	0.98785 (16)	0.87392 (12)	0.0600 (7)	
H41A	0.9706	0.9594	0.9152	0.072*	
C42	0.8979 (2)	0.94048 (14)	0.81760 (12)	0.0581 (7)	
H42A	0.8900	0.8797	0.8217	0.070*	
N10	0.89519 (16)	0.71939 (12)	0.86786 (10)	0.0501 (5)	
O10	0.9058 (2)	0.76082 (15)	0.92176 (12)	0.0773 (7)	
O11	0.95399 (15)	0.72583 (13)	0.83381 (10)	0.0598 (4)	
O12	0.82643 (18)	0.67101 (17)	0.84712 (13)	0.0789 (7)	
O2W	0.88021 (19)	1.31295 (18)	0.57881 (14)	0.0829 (7)	
H1O2	0.9248	1.2790	0.5993	0.124*	
H2O2	0.8795	1.3574	0.6043	0.124*	

### Atomic displacement parameters (Å^2^)

**Table:**

	*U*^11^	*U*^22^	*U*^33^	*U*^12^	*U*^13^	*U*^23^
Cu2	0.0591 (2)	0.03360 (16)	0.04541 (18)	−0.00919 (13)	0.00277 (14)	−0.00994 (12)
Cl1	0.0598 (3)	0.0456 (3)	0.0432 (3)	−0.0050 (2)	−0.0026 (2)	−0.0097 (2)
N1	0.0531 (10)	0.0368 (8)	0.0399 (9)	−0.0078 (7)	0.0070 (7)	−0.0066 (7)
N2	0.0427 (9)	0.0328 (8)	0.0403 (8)	−0.0054 (6)	0.0061 (7)	−0.0063 (6)
N3	0.0569 (11)	0.0337 (9)	0.0557 (11)	−0.0063 (8)	0.0088 (9)	−0.0081 (8)
N8	0.0534 (11)	0.0426 (9)	0.0453 (9)	−0.0022 (8)	0.0080 (8)	0.0020 (8)
O4	0.0515 (14)	0.0482 (12)	0.0612 (13)	−0.0112 (10)	0.0094 (13)	−0.0154 (11)
O5	0.087 (2)	0.109 (3)	0.0670 (17)	−0.007 (2)	0.0153 (15)	−0.0462 (18)
O6	0.0576 (15)	0.102 (2)	0.0740 (18)	0.0114 (14)	0.0004 (12)	−0.0151 (17)
N9	0.0623 (12)	0.0377 (9)	0.0518 (11)	0.0028 (8)	0.0076 (9)	−0.0060 (8)
O7	0.0622 (11)	0.0582 (10)	0.0585 (10)	−0.0155 (9)	0.0115 (8)	−0.0215 (8)
O8	0.0637 (13)	0.0821 (15)	0.0933 (17)	−0.0165 (11)	0.0161 (12)	−0.0364 (13)
O9	0.1051 (18)	0.0619 (11)	0.0688 (13)	0.0017 (12)	0.0344 (13)	−0.0190 (10)
C1	0.0409 (9)	0.0363 (9)	0.0351 (9)	−0.0053 (8)	0.0070 (7)	−0.0054 (7)
C2	0.0529 (12)	0.0362 (10)	0.0401 (10)	−0.0032 (8)	0.0077 (9)	−0.0046 (8)
C3	0.0590 (13)	0.0422 (10)	0.0438 (11)	0.0008 (10)	0.0091 (10)	0.0024 (9)
C4	0.0574 (13)	0.0544 (12)	0.0375 (10)	−0.0024 (10)	0.0025 (9)	0.0001 (9)
C5	0.0618 (14)	0.0484 (12)	0.0392 (10)	−0.0106 (10)	0.0041 (9)	−0.0075 (9)
C6	0.0406 (9)	0.0320 (9)	0.0385 (9)	−0.0029 (7)	0.0098 (8)	−0.0042 (7)
C7	0.0451 (10)	0.0288 (8)	0.0386 (9)	−0.0036 (7)	0.0081 (8)	−0.0026 (7)
C8	0.0385 (9)	0.0334 (9)	0.0384 (9)	−0.0009 (7)	0.0099 (7)	−0.0029 (7)
C9	0.0481 (11)	0.0320 (9)	0.0420 (10)	−0.0012 (8)	0.0077 (8)	−0.0011 (8)
C10	0.0436 (10)	0.0324 (9)	0.0439 (10)	−0.0026 (7)	0.0119 (8)	−0.0028 (8)
C11	0.0494 (11)	0.0320 (9)	0.0524 (12)	−0.0045 (8)	0.0154 (9)	−0.0055 (8)
C12	0.0636 (14)	0.0359 (10)	0.0596 (13)	−0.0048 (10)	0.0174 (11)	−0.0009 (9)
C13	0.0750 (17)	0.0344 (11)	0.0802 (18)	−0.0018 (11)	0.0273 (14)	0.0056 (11)
C14	0.0738 (18)	0.0338 (10)	0.089 (2)	−0.0121 (11)	0.0250 (15)	−0.0106 (12)
C15	0.0674 (16)	0.0380 (11)	0.0737 (17)	−0.0128 (11)	0.0138 (13)	−0.0144 (11)
C16	0.0410 (10)	0.0314 (9)	0.0340 (9)	−0.0005 (7)	0.0085 (7)	−0.0031 (7)
C17	0.0557 (12)	0.0332 (9)	0.0364 (9)	−0.0034 (8)	0.0031 (8)	0.0030 (7)
C18	0.0590 (13)	0.0297 (9)	0.0438 (11)	−0.0059 (8)	0.0056 (9)	−0.0010 (8)
C19	0.0424 (10)	0.0383 (9)	0.0355 (9)	−0.0029 (8)	0.0066 (8)	−0.0080 (8)
C20	0.0549 (12)	0.0369 (9)	0.0392 (10)	0.0057 (9)	0.0013 (9)	−0.0009 (8)
C21	0.0555 (12)	0.0293 (8)	0.0435 (11)	0.0024 (8)	0.0036 (9)	−0.0019 (8)
Cu1	0.04705 (17)	0.03035 (15)	0.03209 (15)	−0.00569 (11)	0.00088 (12)	−0.00446 (10)
Cl2	0.0935 (5)	0.0550 (3)	0.0464 (3)	−0.0186 (3)	−0.0019 (3)	−0.0200 (2)
O1	0.0582 (9)	0.0499 (8)	0.0403 (7)	−0.0183 (7)	0.0030 (7)	−0.0090 (6)
O1W	0.0907 (16)	0.0972 (16)	0.0499 (10)	−0.0037 (13)	0.0288 (11)	−0.0108 (11)
O2	0.0825 (17)	0.109 (2)	0.0879 (17)	−0.0557 (16)	0.0212 (13)	−0.0209 (15)
O3	0.0870 (15)	0.0717 (12)	0.0667 (12)	−0.0139 (12)	0.0270 (11)	−0.0181 (11)
N4	0.0477 (9)	0.0302 (7)	0.0378 (8)	−0.0016 (7)	0.0077 (7)	−0.0039 (6)
N5	0.0407 (8)	0.0288 (7)	0.0316 (7)	−0.0058 (6)	0.0052 (6)	−0.0038 (6)
N6	0.0441 (9)	0.0357 (8)	0.0340 (8)	−0.0032 (7)	0.0024 (7)	0.0001 (6)
N7	0.0574 (12)	0.0500 (10)	0.0480 (10)	−0.0135 (9)	0.0046 (9)	−0.0022 (8)
C22	0.0426 (10)	0.0305 (8)	0.0335 (8)	−0.0029 (7)	0.0096 (7)	−0.0039 (7)
C23	0.0537 (12)	0.0358 (10)	0.0399 (10)	0.0015 (8)	0.0061 (9)	−0.0002 (8)
C24	0.0642 (14)	0.0341 (10)	0.0576 (13)	0.0077 (10)	0.0093 (11)	0.0042 (9)
C25	0.0712 (15)	0.0283 (9)	0.0620 (14)	0.0017 (9)	0.0132 (12)	−0.0073 (9)
C26	0.0590 (13)	0.0337 (9)	0.0483 (11)	−0.0052 (9)	0.0070 (9)	−0.0126 (8)
C27	0.0400 (9)	0.0312 (8)	0.0302 (8)	−0.0028 (7)	0.0068 (7)	−0.0005 (7)
C28	0.0442 (10)	0.0303 (8)	0.0309 (8)	−0.0016 (7)	0.0041 (7)	−0.0004 (7)
C29	0.0421 (9)	0.0296 (8)	0.0320 (8)	−0.0031 (7)	0.0059 (7)	−0.0023 (7)
C30	0.0450 (10)	0.0266 (8)	0.0361 (9)	−0.0025 (7)	0.0063 (8)	−0.0014 (7)
C31	0.0403 (9)	0.0300 (8)	0.0329 (9)	−0.0038 (7)	0.0080 (7)	−0.0004 (7)
C32	0.0374 (9)	0.0346 (9)	0.0323 (8)	−0.0042 (7)	0.0073 (7)	0.0006 (7)
C33	0.0419 (10)	0.0343 (9)	0.0379 (9)	−0.0022 (7)	0.0089 (8)	0.0016 (7)
C34	0.0446 (11)	0.0417 (10)	0.0413 (10)	0.0035 (8)	0.0103 (8)	0.0082 (8)
C35	0.0460 (11)	0.0534 (12)	0.0353 (9)	0.0004 (9)	0.0024 (8)	0.0058 (9)
C36	0.0486 (11)	0.0452 (10)	0.0367 (10)	−0.0044 (9)	0.0023 (8)	−0.0023 (8)
C37	0.0461 (10)	0.0297 (8)	0.0328 (9)	−0.0018 (7)	0.0030 (7)	−0.0045 (7)
C38	0.0582 (12)	0.0320 (9)	0.0377 (10)	−0.0035 (8)	0.0016 (9)	−0.0008 (8)
C39	0.0680 (15)	0.0315 (9)	0.0499 (12)	−0.0091 (9)	0.0062 (11)	−0.0082 (9)
C40	0.0561 (12)	0.0407 (10)	0.0400 (10)	−0.0065 (9)	0.0040 (9)	−0.0129 (8)
C41	0.0896 (19)	0.0403 (11)	0.0364 (10)	0.0035 (12)	−0.0073 (11)	−0.0033 (9)
C42	0.097 (2)	0.0281 (9)	0.0376 (11)	−0.0009 (11)	−0.0034 (11)	−0.0025 (8)
N10	0.0618 (12)	0.0311 (8)	0.0489 (10)	−0.0042 (8)	−0.0005 (9)	0.0087 (7)
O10	0.1122 (19)	0.0648 (12)	0.0536 (11)	−0.0207 (12)	0.0193 (11)	−0.0108 (9)
O11	0.0646 (11)	0.0525 (9)	0.0594 (10)	0.0011 (8)	0.0113 (9)	0.0076 (8)
O12	0.0731 (14)	0.0745 (13)	0.0776 (14)	−0.0272 (12)	0.0000 (11)	0.0053 (11)
O2W	0.0724 (14)	0.0842 (15)	0.0845 (15)	0.0128 (12)	0.0073 (12)	0.0130 (13)

### Geometric parameters (Å, °)

**Table:**

Cu2—N2	1.9256 (18)	Cu1—O1	1.9806 (16)
Cu2—N1	2.0096 (19)	Cu1—N4	2.0178 (18)
Cu2—N3	2.014 (2)	Cu1—N6	2.0277 (18)
Cu2—O7	2.0475 (18)	Cu1—O1W	2.208 (2)
Cu2—O4	2.163 (3)	Cl2—C40	1.739 (2)
Cl1—C19	1.742 (2)	O1—N7	1.297 (3)
N1—C5	1.326 (3)	O1W—H1O1	0.8500
N1—C1	1.352 (3)	O1W—H2O1	0.8501
N2—C10	1.333 (3)	O2—N7	1.218 (3)
N2—C6	1.336 (3)	O3—N7	1.231 (3)
N3—C15	1.339 (3)	N4—C26	1.334 (3)
N3—C11	1.354 (3)	N4—C22	1.359 (3)
N8—O5	1.202 (3)	N5—C31	1.332 (3)
N8—O4'	1.214 (14)	N5—C27	1.338 (3)
N8—O6'	1.222 (14)	N6—C36	1.339 (3)
N8—O6	1.240 (4)	N6—C32	1.353 (3)
N8—O5'	1.263 (13)	C22—C23	1.379 (3)
N8—O4	1.279 (3)	C22—C27	1.488 (2)
N9—O8	1.229 (3)	C23—C24	1.394 (3)
N9—O9	1.232 (3)	C23—H23A	0.9300
N9—O7	1.267 (3)	C24—C25	1.378 (4)
C1—C2	1.384 (3)	C24—H24A	0.9300
C1—C6	1.485 (3)	C25—C26	1.378 (4)
C2—C3	1.394 (3)	C25—H25A	0.9300
C2—H2A	0.9300	C26—H26A	0.9300
C3—C4	1.372 (3)	C27—C28	1.382 (3)
C3—H3A	0.9300	C28—C29	1.407 (3)
C4—C5	1.389 (4)	C28—H28A	0.9300
C4—H4A	0.9300	C29—C30	1.400 (3)
C5—H5A	0.9300	C29—C37	1.486 (3)
C6—C7	1.386 (3)	C30—C31	1.385 (3)
C7—C8	1.403 (3)	C30—H30A	0.9300
C7—H7A	0.9300	C31—C32	1.492 (2)
C8—C9	1.404 (3)	C32—C33	1.383 (3)
C8—C16	1.490 (3)	C33—C34	1.395 (3)
C9—C10	1.385 (3)	C33—H33A	0.9300
C9—H9A	0.9300	C34—C35	1.373 (3)
C10—C11	1.489 (3)	C34—H34A	0.9300
C11—C12	1.375 (4)	C35—C36	1.384 (3)
C12—C13	1.396 (3)	C35—H35A	0.9300
C12—H12A	0.9300	C36—H36A	0.9300
C13—C14	1.370 (5)	C37—C42	1.387 (3)
C13—H13A	0.9300	C37—C38	1.392 (3)
C14—C15	1.374 (4)	C38—C39	1.389 (3)
C14—H14A	0.9300	C38—H38A	0.9300
C15—H15A	0.9300	C39—C40	1.372 (3)
C16—C21	1.390 (3)	C39—H39A	0.9300
C16—C17	1.399 (3)	C40—C41	1.377 (3)
C17—C18	1.383 (3)	C41—C42	1.379 (3)
C17—H17A	0.9300	C41—H41A	0.9300
C18—C19	1.378 (3)	C42—H42A	0.9300
C18—H18A	0.9300	N10—O12	1.228 (3)
C19—C20	1.383 (3)	N10—O10	1.239 (3)
C20—C21	1.386 (3)	N10—O11	1.249 (3)
C20—H20A	0.9300	O2W—H1O2	0.8500
C21—H21A	0.9300	O2W—H2O2	0.8501
Cu1—N5	1.9287 (16)		
			
N2—Cu2—N1	80.40 (8)	C21—C20—H20A	120.6
N2—Cu2—N3	80.07 (8)	C20—C21—C16	121.70 (19)
N1—Cu2—N3	160.46 (8)	C20—C21—H21A	119.2
N2—Cu2—O7	146.70 (8)	C16—C21—H21A	119.2
N1—Cu2—O7	102.01 (8)	N5—Cu1—O1	154.52 (7)
N3—Cu2—O7	94.10 (9)	N5—Cu1—N4	80.39 (7)
N2—Cu2—O4	132.92 (9)	O1—Cu1—N4	96.69 (7)
N1—Cu2—O4	92.52 (11)	N5—Cu1—N6	79.97 (7)
N3—Cu2—O4	101.09 (11)	O1—Cu1—N6	101.54 (7)
O7—Cu2—O4	80.36 (9)	N4—Cu1—N6	160.33 (7)
C5—N1—C1	119.73 (19)	N5—Cu1—O1W	118.45 (9)
C5—N1—Cu2	125.54 (15)	O1—Cu1—O1W	86.84 (9)
C1—N1—Cu2	114.59 (15)	N4—Cu1—O1W	91.94 (9)
C10—N2—C6	121.93 (18)	N6—Cu1—O1W	96.37 (9)
C10—N2—Cu2	119.28 (14)	N7—O1—Cu1	110.17 (13)
C6—N2—Cu2	118.61 (15)	Cu1—O1W—H1O1	120.7
C15—N3—C11	118.7 (2)	Cu1—O1W—H2O1	128.1
C15—N3—Cu2	126.5 (2)	H1O1—O1W—H2O1	107.3
C11—N3—Cu2	114.79 (14)	C26—N4—C22	118.74 (19)
O5—N8—O4'	138.8 (13)	C26—N4—Cu1	126.75 (16)
O5—N8—O6'	92.3 (14)	C22—N4—Cu1	114.48 (13)
O4'—N8—O6'	128.3 (19)	C31—N5—C27	121.60 (16)
O5—N8—O6	123.3 (3)	C31—N5—Cu1	119.44 (14)
O4'—N8—O6	97.3 (13)	C27—N5—Cu1	118.77 (13)
O6'—N8—O6	31.1 (13)	C36—N6—C32	119.07 (18)
O5—N8—O5'	28.7 (9)	C36—N6—Cu1	126.56 (15)
O4'—N8—O5'	113.3 (16)	C32—N6—Cu1	114.36 (13)
O6'—N8—O5'	118.3 (17)	O2—N7—O3	124.2 (3)
O6—N8—O5'	148.4 (10)	O2—N7—O1	118.0 (2)
O5—N8—O4	120.0 (3)	O3—N7—O1	117.7 (2)
O4'—N8—O4	20.7 (12)	N4—C22—C23	121.93 (18)
O6'—N8—O4	147.7 (14)	N4—C22—C27	113.44 (17)
O6—N8—O4	116.7 (3)	C23—C22—C27	124.62 (18)
O5'—N8—O4	93.0 (10)	C22—C23—C24	118.4 (2)
N8—O4—Cu2	113.81 (19)	C22—C23—H23A	120.8
O8—N9—O9	121.8 (3)	C24—C23—H23A	120.8
O8—N9—O7	118.8 (2)	C25—C24—C23	119.5 (2)
O9—N9—O7	119.3 (2)	C25—C24—H24A	120.2
N9—O7—Cu2	111.52 (14)	C23—C24—H24A	120.2
N1—C1—C2	121.5 (2)	C26—C25—C24	118.9 (2)
N1—C1—C6	113.54 (18)	C26—C25—H25A	120.6
C2—C1—C6	124.92 (18)	C24—C25—H25A	120.6
C1—C2—C3	118.3 (2)	N4—C26—C25	122.5 (2)
C1—C2—H2A	120.8	N4—C26—H26A	118.7
C3—C2—H2A	120.8	C25—C26—H26A	118.7
C4—C3—C2	119.7 (2)	N5—C27—C28	120.86 (17)
C4—C3—H3A	120.1	N5—C27—C22	112.81 (16)
C2—C3—H3A	120.1	C28—C27—C22	126.32 (18)
C3—C4—C5	118.8 (2)	C27—C28—C29	119.05 (18)
C3—C4—H4A	120.6	C27—C28—H28A	120.5
C5—C4—H4A	120.6	C29—C28—H28A	120.5
N1—C5—C4	121.9 (2)	C30—C29—C28	118.38 (17)
N1—C5—H5A	119.1	C30—C29—C37	121.79 (17)
C4—C5—H5A	119.1	C28—C29—C37	119.83 (17)
N2—C6—C7	120.35 (19)	C31—C30—C29	119.24 (17)
N2—C6—C1	112.63 (18)	C31—C30—H30A	120.4
C7—C6—C1	127.02 (18)	C29—C30—H30A	120.4
C6—C7—C8	119.44 (17)	N5—C31—C30	120.84 (18)
C6—C7—H7A	120.3	N5—C31—C32	112.28 (17)
C8—C7—H7A	120.3	C30—C31—C32	126.88 (17)
C7—C8—C9	118.33 (19)	N6—C32—C33	121.96 (18)
C7—C8—C16	120.55 (17)	N6—C32—C31	113.75 (17)
C9—C8—C16	121.11 (19)	C33—C32—C31	124.28 (18)
C10—C9—C8	119.0 (2)	C32—C33—C34	118.45 (19)
C10—C9—H9A	120.5	C32—C33—H33A	120.8
C8—C9—H9A	120.5	C34—C33—H33A	120.8
N2—C10—C9	120.90 (18)	C35—C34—C33	119.3 (2)
N2—C10—C11	112.43 (19)	C35—C34—H34A	120.4
C9—C10—C11	126.6 (2)	C33—C34—H34A	120.4
N3—C11—C12	122.0 (2)	C34—C35—C36	119.5 (2)
N3—C11—C10	113.4 (2)	C34—C35—H35A	120.3
C12—C11—C10	124.6 (2)	C36—C35—H35A	120.3
C11—C12—C13	118.3 (3)	N6—C36—C35	121.8 (2)
C11—C12—H12A	120.9	N6—C36—H36A	119.1
C13—C12—H12A	120.9	C35—C36—H36A	119.1
C14—C13—C12	119.7 (3)	C42—C37—C38	117.65 (19)
C14—C13—H13A	120.2	C42—C37—C29	120.71 (18)
C12—C13—H13A	120.2	C38—C37—C29	121.64 (18)
C13—C14—C15	118.9 (2)	C39—C38—C37	120.9 (2)
C13—C14—H14A	120.5	C39—C38—H38A	119.6
C15—C14—H14A	120.5	C37—C38—H38A	119.6
N3—C15—C14	122.4 (3)	C40—C39—C38	119.6 (2)
N3—C15—H15A	118.8	C40—C39—H39A	120.2
C14—C15—H15A	118.8	C38—C39—H39A	120.2
C21—C16—C17	117.73 (18)	C39—C40—C41	121.0 (2)
C21—C16—C8	121.72 (18)	C39—C40—Cl2	120.39 (17)
C17—C16—C8	120.54 (18)	C41—C40—Cl2	118.63 (19)
C18—C17—C16	121.35 (19)	C40—C41—C42	118.9 (2)
C18—C17—H17A	119.3	C40—C41—H41A	120.6
C16—C17—H17A	119.3	C42—C41—H41A	120.6
C19—C18—C17	119.19 (18)	C41—C42—C37	122.1 (2)
C19—C18—H18A	120.4	C41—C42—H42A	119.0
C17—C18—H18A	120.4	C37—C42—H42A	119.0
C18—C19—C20	121.20 (19)	O12—N10—O10	120.3 (3)
C18—C19—Cl1	119.47 (16)	O12—N10—O11	118.9 (2)
C20—C19—Cl1	119.33 (17)	O10—N10—O11	120.8 (2)
C19—C20—C21	118.8 (2)	H1O2—O2W—H2O2	107.7
C19—C20—H20A	120.6		
